# Facial-submental island flap for reconstruction of hemitongue defects in young, middle-aged and elderly patients with early and middle stage oral tongue squamous cell carcinoma

**DOI:** 10.1186/s13005-022-00343-0

**Published:** 2022-12-05

**Authors:** Yan Wang, Bin Zhou, Wei-liang Chen, Zi-xian Huang, Rui Chen

**Affiliations:** Department of Oral and Maxillofacial Surgery, Sun Yat-sen Memorial Hospital, Sun Yat-sen University, 107 Yan-jiang Road, Guangzhou, 510120 China

**Keywords:** Tongue cancer, Tongue defects, Submental flap, Young, middle-aged, and elderly patients, Comorbidities, Adult comorbidity Evaluation-27, Complications, Clavien-Dindo classification, Early and middle-stage

## Abstract

**Background:**

This study evaluated the outcomes of facial-submental artery island flap (FSAIF) for reconstruction of the hemitongue following cancer ablation in patients with early and middle-stage oral tongue squamous cell carcinoma (OTSCC).

**Methods:**

In total, 122 patients with early and middle-stage OTSCC were divided into young, middle-aged, and elderly groups. The Adult Comorbidity Evaluation-27 (ACE-27) index was used to determine the presence of comorbidities. The patients underwent surgical treatment with hemiglossectomy, neck dissection, and hemitongue reconstruction using FSAIF. In addition, stage I (*n* = 15) and II (*n* = 69) patients underwent ipsilateral selective neck dissection, whereas those with stage III (*n* = 38) underwent radical neck dissection. Six patients with T3N1 disease also underwent cobalt-60 adjuvant radiotherapy.

**Results:**

Young and elderly patients exhibited significant differences in comorbidities, as assessed by the ACE-27 (*p* < .05). The skin paddles in the young, middle-aged, and elderly patients were 3 × 9 to 4 × 12 cm, 3 × 11 to 4 × 12, and 3 ×  10 to 5 × 13 cm in size, respectively. FSAIF failure occurred in four patients (success rate: 96.7%). No significant differences were observed in the skin paddle of the flap or rate of flap failure among the age groups (*p* > .05). Clavien-Dindo grades I, II, IIIa, IIIb, Iva, and IVb were assigned to 7.1, 36.1, 38.5, 9.8, 4.1, and 4.1% of the patients, respectively, with significant differences seen between the young and elderly patients (*p* < .05). In total, 52.5% of patients could eat normally, whereas 32.8% required a soft diet. Furthermore, 53.3 and 33.6% of patients achieved normal and intelligible speech, respectively. The aesthetic results were rated as excellent and good in 32.8 and 58.2% of patients, respectively. In total, 68.0% of the patients were alive and exhibited no evidence of disease, while 19.7% were alive with active disease. In addition, 12.3% of patients with stage III OTSCC died due to local recurrence or distant metastases. No differences in swallowing, speech, aesthetic, or survival outcomes were observed among the groups.

**Conclusions:**

FSAIF is a simple, safe, and reliable method for reconstructing hemitongue defects following cancer ablation in young, middle-aged, and elderly patients with early and middle-stage OTSCC.

## Introduction

Oral tongue squamous cell carcinoma (OTSCC) is the most common primary tumor of the oral cavity; it accounts for 87% of all cases of oral SCC [1]. OTSCC predominantly affects older adults, but its prevalence in younger patients is increasing worldwide [2]. Younger patients are often diagnosed at a later stage of cancer than older patients, and have higher rates of regional metastases and delayed relapse. Furthermore, recurrent disease is more aggressive than the initial disease [3]. A study of multihospital claims database from > 1000 patients aged < 45 years reported that tongue cancer was not associated with a poor prognosis [4]. In addition, treatment decision-making is particularly challenging in older patients with comorbidities, such as cardiovascular, endocrine, and musculoskeletal diseases. The Adult Comorbidity Evaluation-27 (ACE-27) [5] is used to assess comorbidities and correlates with the overall survival of head and neck cancer patients older than 70 years [6]. Several factors are associated with poor outcomes in elderly OTSCC patients, including their clinicopathological characteristics and surgical management [7]. It is unclear whether the prognosis of OTSCC differs between young and elderly patients. We previously reported that the facial-submental artery island flap (FSAIF) can be reliably used to reconstruct oral and maxillofacial defects following cancer ablation [8], particularly in older patients [9]. In the present study, we evaluated the outcomes of hemitongue reconstruction using FSAIF following cancer ablation in young, middle-aged, and elderly patients with early and middle-stage OTSCC.

### Patients and methods

This retrospective observational study was conducted from June 2011 to May 2021 at the Department of Oral and Maxillofacial Surgery, Sun Yat-sen Memorial Hospital, Sun Yat-sen University, China. The Institutional Review Board of Sun Yat-sen University approved the study. Data on age, sex, comorbidities, clinical staging, flap size, length of surgery, flap survival, complications, swallowing and speech functions, aesthetic outcome, and survival status were extracted from patients’ medical records. The study included primary T1–T3 stage OTSCC patients; there were no N0 stage patients. We excluded patients with cachexia, severe congestive cardiac failure, severe chronic obstructive pulmonary disease, and/or missing follow-up data.

**Fig. 1:**
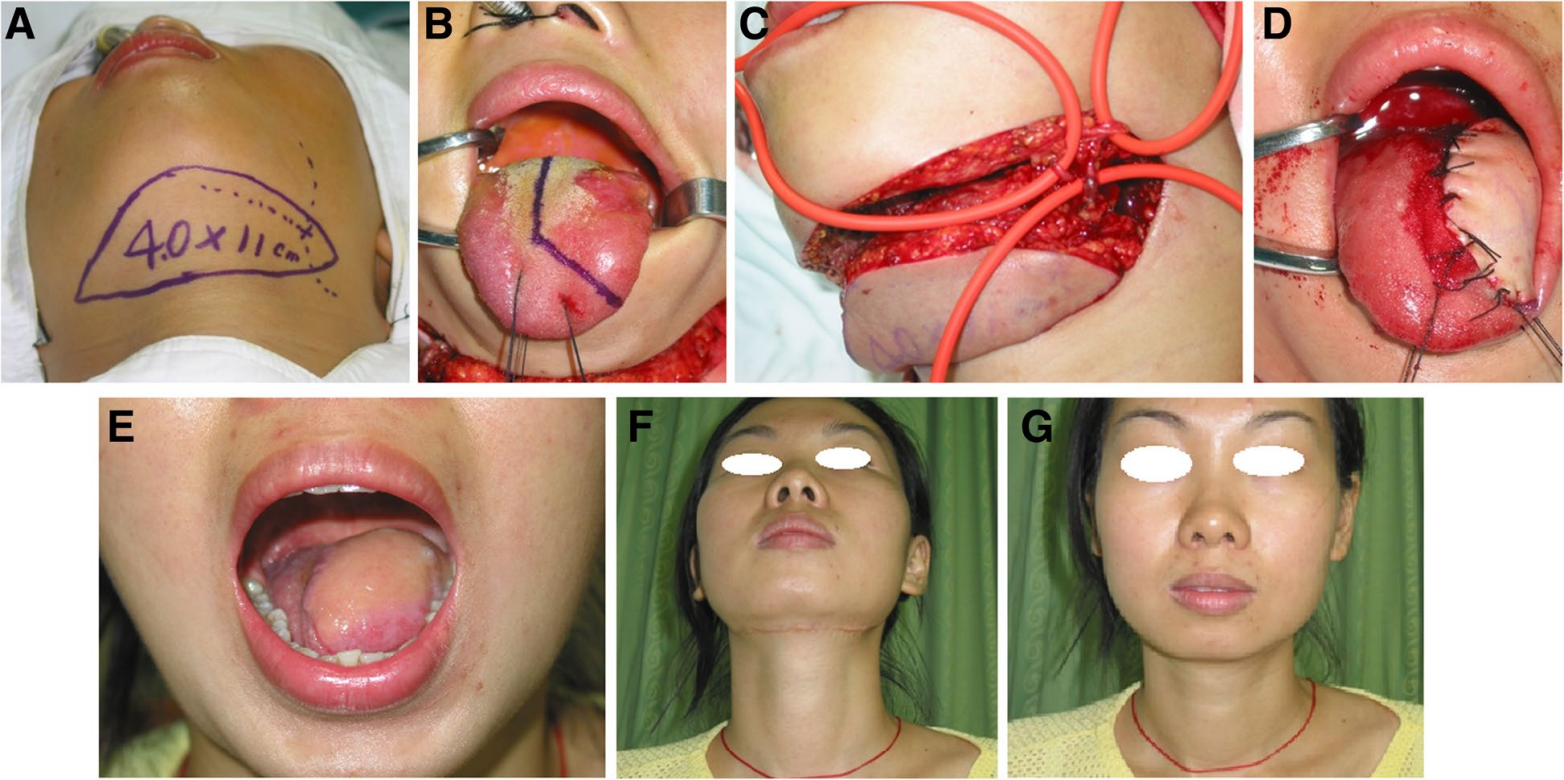
A 23-year-old female patient with stage II oral tongue squamous cell carcinoma (OTSCC). Incision for the submental artery island flap (FASIF) (**A**) and the tongue tumor (**B**). Flap harvested (**C**) through FASIF reconstruction of the hemitongue (**D**). Hemitongue reconstruction (**E**) and a well-hidden horizontal scar at the donor site (F, **G**) at 18 months postoperatively

**Fig. 2:**
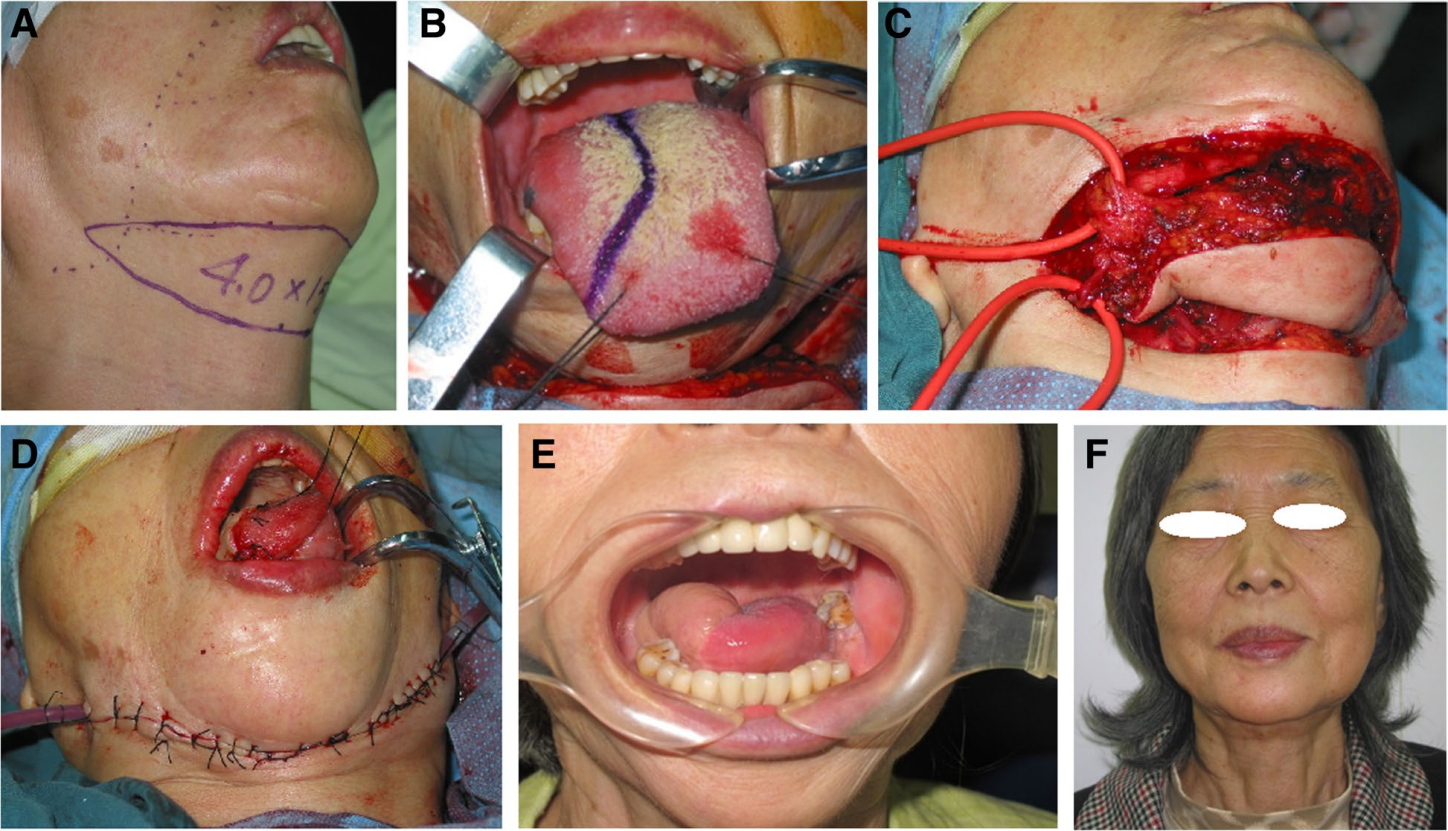
A 64-year-old female patient with stage II OTSCC. Incision for FASIF (**A**) and tongue tumor (**B**). The harvested flap (**C**). FASIF reconstruction of the hemitongue. The donor area was largely closed (**D**). Hemitongue reconstruction (**E**) and a well-hidden horizontal scar (**F**) at 60 months postoperatively

**Fig. 3:**
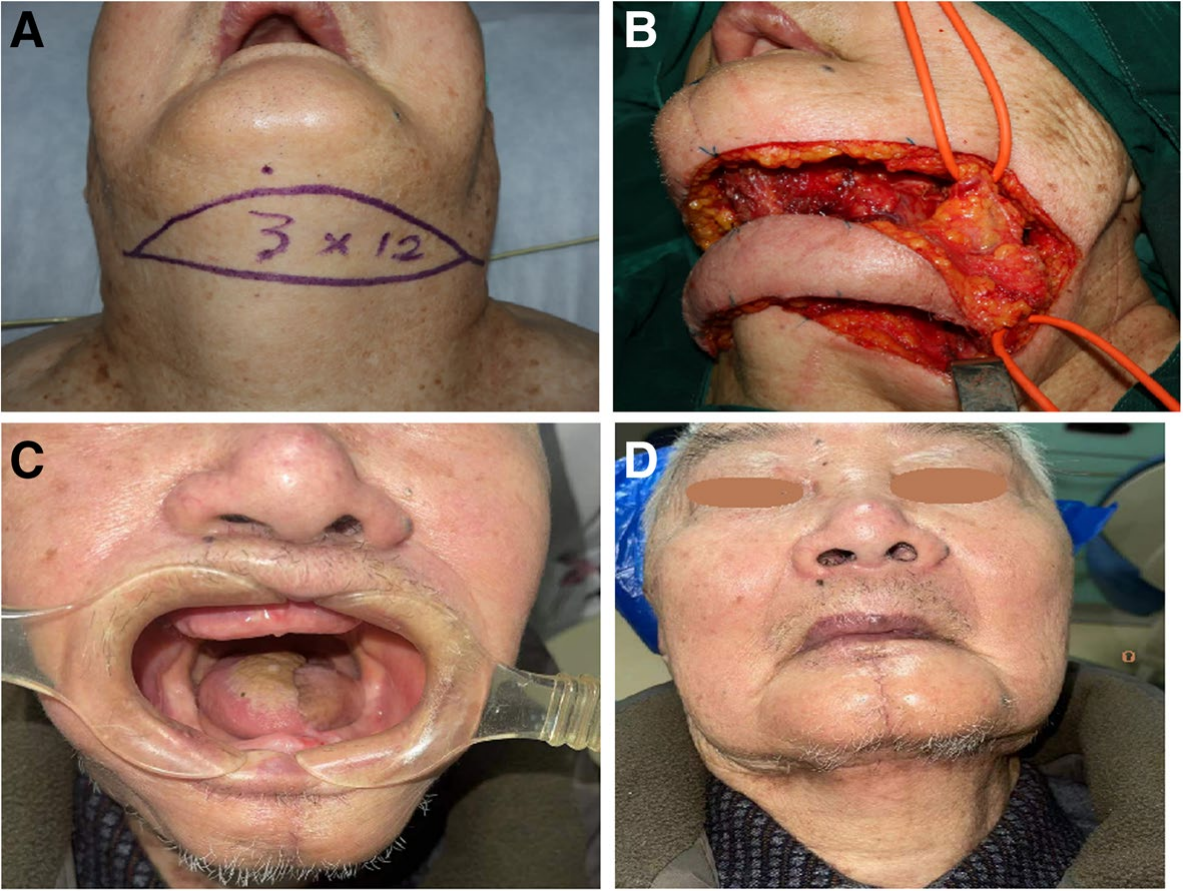
A 90-year-old male patient with stage II OTSCC. Incision plan for FASIF (**A**) and the harvested flap (**B**). Hemitongue reconstruction (**C**) leaving a well-hidden horizontal scar (**E**) at 16 months postoperatively

We enrolled 122 OTSCC patients (64 males [52.5%] and 58 females [47.5%]) aged 23–90 years (median age, 58.3 years). The patients were categorized as young (< 45 years, *n* = 18, 14.8%) (Fig. 1), middle-aged (≥ 45 to < 65 years, *n* = 63, 51.6%) (Fig. 2), or elderly (≥ 65 years, *n* = 41, 33.6%) (Fig. 3). The 8th edition of the American Joint Committee on Cancer (AJCC) staging manual [10] was used for the clinical staging of OTSCC. Int total, 3, 11, and 4 patients in the young group, 8, 36, and 19 patients in the middle-aged group, and 4, 22, and 15 in the elderly group were classified as stage I (T1N0), II (T2N0), and III (T3N0 and T3N1), respectively.

The ACE-27 index was used to evaluate comorbidities including alcohol abuse, hypertension, respiratory disease, congestive heart failure, diabetes mellitus, arrhythmia, myocardial infarction, coronary artery disease/angina, stroke, renal insufficiency, dementia, paralysis, other solid tumors, obesity, leukemia/myeloma, lymphoma, illicit drug use, and peripheral arterial, gastrointestinal, psychiatric, liver, neuromuscular, venous, pancreatic, rheumatological, and immunological (e.g., AIDS) diseases. The diseases and conditions were categorized into grades 1–3 (mild, moderate, and severe, respectively) according to the severity of organ decompensation and prognosis. Based on the highest-ranked ailment among the diseases and comorbidities, we assigned patients to comorbidity classes (none, mild, moderate, or severe). Patients with two or more moderate ailments affecting different organ systems, or belonging to different groups, were classified into the severe comorbidities group [11]. The comorbidities were extracted from the medical records, as stated above. In total, 67 (54.9%) patients had at least one comorbidity. Based on the ACE-27 index, 37 (30.3%), 20 (16.4%), and 10 (8.2%) patients had mild, moderate, and severe comorbidities, respectively.

Surgery, including hemiglossectomy, ipsilateral selective neck dissection, and hemitongue reconstruction using FSAIF, was performed in 122 patients. Ipsilateral selective neck dissection was performed in 15 and 69 patients with stage I and II disease, respectively, and radical neck dissection was performed in 38 patients with stage III disease. The submandibular lymph nodes were carefully removed and subjected to the rapid pathological diagnosis during flap elevation. Then, the donor area was largely closed. The details of the surgical technique have been described previously [8]. Six patients with T3N1 disease underwent cobalt-60 adjuvant radiotherapy (60 Gy over 30 days; 2 Gy fractions/day) at the primary site. Table 1 presents the patients’ demographic and clinical characteristics.

**Table 1:** Demographics, clinical characteristics, and outcomes of facial-submental island flap for reconstruction of tongue defects following cancer ablation in young (< 45 years), middle-aged (≥ 45 to < 65 years) and elderly (≥ 65 years age) patients with early and middle stage oral tongue squamous cell carcinoma

	Young group (***n*** = 18) No. of patients (%)	Middle-aged group (***n*** = 63) No. of patients (%)	Elderly group (***n*** = 41) No. of patients (%)	***P***-value
**Age range, years** (mean ± SD)	23–44, 48.6 ± 8.69	45–64, 55.4 ± 9.6	65–90, 71.9 ± 13.9	**< .014**
**Sex**				**.654**
Male	10 (55.6)	29 (46.0)	25 (61.0)	
Female	8 (44.4)	34 (54.0)	16 (39.0)	
**AJCC cancer stage**				.763
Stage I T1N0	3 (11.7)	8 (12.7)	4 (9.8)	
Stage II T2N0	11 (66.1)	36 (57.1)	22 (53.6)	
Stage III (T3N0 + T3N1)	3 + 1 (22.2)	16 + 3 (30.2)	13 + 2 (36.6)	
**ACE-27**				**.036**^**※**^
None	14 (77.8)	33 (52.4)	8 (19.5)	
Mild	3 (16.6)	18 (28.6)	16 (39.0)	
Moderate	1 (5.6)	10 (15.9)	9 (22.0)	
Severe	0 (0.0)	2 (3.1)	8 (19.5)	
**Neck dissection**				**.789**
Selective	14 (77.8)	44 (69.8)	26 (63.4)	
Radical	4 (22.2)	19 (30.2)	15 (36.6)	
**Paddle size**				**.862**
Range, median (cm)	3 × 9–4 × 12, 3.6× 10.3	3 × 11–4 × 15, 3.8 × 13.6	3 × 10–5 × 13, 3.7 × 12.2	
**Successes (no.**	18 (100.0)	61 (96.8)	39 (95.1)	**.783**
**Length of surgery**				**.699**
**<** 120 minutes	14 (77.8)	45 (71.4)	26 (63.4)	
**≥** 120 minutes	4 (22.2)	18 (28.6)	15 (36.6)	
**Clavien-Dindo classification**				**.029**^**※**^
I	4 (22.2)	4 (7.9)	1 (2.4)	
II	9 (50.0)	23 (35.0)	12 (29.3)	
IIIa	4 (22.2)	26 (41.3)	17 (41.5)	
IIIb	1 (5.6)	6 (9.4)	5 (12.2)	
IVa	0 (0.0)	2 (3.2)	3 (7.3)	
IVb	0 (0.0)	2 (3.2)	3 (7.3)	
**Swallowing functions**				**.664**
Normal	10 (55.6)	34 (54.0)	20 (48.8)	
Soft	6 (33.3)	21 (33.3)	13 (31.7)	
Liquid	2 (11.1)	8 (12.7)	8 (19.5)	
**Speech functions**				**.559**
Normal	11 (61.1)	35 (55.6)	19 (46.3)	
Intelligible	6 (33.3)	22 (34.9)	13 (31.7)	
Slurred	1 (5.6)	6 (9.5)	9 (22.0)	
**Aesthetic outcome**				**.564**
Excellent	5 (27.7)	20 (31.8)	15 (36.6)	
Good	10 (55.6)	37 (58.7)	13 (58.6)	
Fair	3 (16.7)	6 (9.5)	8 (4.8)	
Adjuvant radiotherapy				**.846**
No	17 (94.4)	60 (95.2)	39 (95.1)	
Yes	1 (5.6)	3 (4.8)	2 (4.9)	
**Follow-up range, median (months)**	7 ~ 72, 35.8	7 ~ 80, 40.9	7 ~ 52, 32.6	**.682**
**Status**				**.594**
Alive with no disease	13 (72.2)	45 (71.4)	25 (61.0)	
Alive with disease	3 (16.7)	12 (19.1)	9 (22.0)	
Died of disease	2 (11.1)	6 (9.5)	7 (17.0)	

AJCC cancer stage: American Joint Committee on Cancer (AJCC) Staging; ACE-27: Adult comorbidity evaluation-27; ^※^*P*-value is between the young group and elderly group groups

Postoperative complications that occurred within 30 days after surgery were assessed using the Clavien-Dindo classification (Table 2) [12]. The patients were followed to determine their swallowing and speech functions at 6 months postoperatively. Three surgeons assessed the outcomes. Swallowing was classified as normal, soft, liquid, or nasogastric tube feeding. Speech was classified as normal, intelligible, slurred, or tracheostomy requirement. Esthetic outcome was classified as excellent, good, fair, or poor.

**Table 2:** Clavien-Dindo Classification System for Surgical Complications

Grade	Definition
I	Any deviation from the normal postoperative course without the need for pharmacological treatment or surgical, endoscopic, or radiological interventions. The permitted therapeutic regimens include drugs, such as antiemetics, antipyretics, analgesics, diuretics, and electrolytes, and physiotherapy. This grade also includes wound infections that required opening at the bedside.
II	Requiring pharmacological treatment with drugs other than those permitted for grade I complications, including blood transfusions and total parenteral nutrition.
III	Requiring surgical, endoscopic, or radiological intervention
IIIa	Intervention not under general anesthesia
IIIb	Intervention under general anesthesia
IV	Life-threatening complications (including those affecting the central nervous system) that require management in a high dependency or intensive therapy unit
IVa	Single organ dysfunction (including dialysis)
IVb	Multiorgan dysfunction
**V**	Death

From: Dindo D, Demartines N, Clavien PA. Classification of surgical complications: A new proposal with evaluation in a cohort of 6336 patients and results of a survey. Ann Surg. 2004; 240 (2):205–13

Statistical analyses were performed using SPSS software (version 20.0; IBM Corp., Armonk, NY, USA). The chi-square test, independent samples t-test, and Mann–Whitney U test were used to analyze the data. The level of significance was set at *p* < .05.

## Results

There were no significant differences in sex or AJCC cancer stage among the young, middle-aged, and elderly patients (*p* > .05). According to the ACE-27 index, there were no comorbidities and at least one comorbidity in 55 (45.1%) and 67 (54.9%) patients, respectively. Among the patients with at least one comorbidity, 37 (30.3%), 20 (16.4%), and 10 (8.2%) had mild, moderate, and severe comorbidities. There were no significant differences in comorbidities or ACE-27 score between the young and elderly patients (*p* < .05). The skin paddles in the young, middle-aged, and elderly patients were 3 × 9 to 4 × 12 cm (median, 3.6 × 10.3 cm), 3 × 11 to 4 × 12 cm (median, 3.8 × 12.6 cm), 3 × 10 to 5 × 13 (median, 3.7 × 12.2 cm) in size, respectively. Four patients had FSAIF failure, such that the overall success rate was 96.7%. No significant difference was observed in the skin paddle of the flap or rate of flap failure among the three age groups (*p* > .05). In total, 69.7% (85/122) of patients had a length of surgery of < 120/min. No difference was observed in the length of surgery among the young, middle-aged, and elderly patients (*p* > .05). Six patients (4.9%) had pathologically confirmed occult cervical lymph node metastasis at level Ib (*n* = 2), IIa (*n* = 3), or III (*n* = 1). Clavien-Dindo classification grades I, II, IIIa, IIIb, IVa, and IVb were assigned to 9 (7.1%), 44 (36.1%), 47 (38.5%), 12 (9.8%), 5 (4.1%), and 5 (4.1%) cases, respectively. No grade V surgical complications were observed. There was a significant difference in the complications rate between the young and elderly patients (*p* < .05). Grade IIIa complications, including bleeding and orocutaneous fistulas, were successfully treated with firm pressure or debridement. Among the 12 patients with grade IIIb complications those with hemorrhage and flap failure were treated via an urgent exploratory operation to control the bleeding, and by removal of the failed flap followed by defect repair using an extensive segmental pectoralis major myocutaneous flap, respectively [13]. Ten (8.2%) patients with grade IVa or IVb complications, including respiratory and cardiovascular diseases, brain hemorrhage, and ischemic stroke, received medical treatment in the intensive care unit.

At 6 months postoperatively, 64 (52.5%) patients could eat normally, 40 (32.8%) could tolerate a soft diet, and 18 (14.75%) could tolerate a liquid; none required tube feeding. In addition, 65 (53.3%), 41 (33.6%), and 16 (13.1%) patients achieved normal, intelligible, and slurred speech, respectively, and none required a permanent tracheostomy. The aesthetic results were rated as excellent, good, fair, and poor in 32.8% (Figs. 1E–G, 2E and F, and 3C and E), 58.2, 9, and 0% of patients, respectively. No differences in swallowing, speech, or aesthetic outcomes were observed among the three groups. The patients were followed for 7–80 months (median, 35.8, 40.9, and 32.6 months in the young, middle-aged, and elderly patients, respectively). At the final follow-up, 83 (68.0%) patients were alive with no evidence of disease, including 13, 45, and 25 patients in the young, middle-aged, and elderly groups, respectively. In addition, 24 (19.7%) patients were alive with disease (3, 12, and 9 in the young, middle-aged, and elderly groups, respectively), which affected the primary site (17 case) or neck (7 cases). Furthermore, 15 (12.3%) patients with stage III OTSCC died of local recurrence or distant metastases (2, 6, and 7 in the young, middle-aged, and elderly groups, respectively). No significant difference in survival was observed among the patient groups. Table 1 presents the outcomes of FSAIF for the reconstruction of tongue defects following cancer ablation in young, middle-aged, and elderly patients with early or middle-stage OTSCC.

## Discussion

The goals of hemitongue reconstruction following cancer ablation are to preserve the mobility of the residual tongue segment, restore its shape and volume, encourage normal swallowing and speech, and extend the patient’s lifespan. Microsurgical techniques are the most commonly used methods for oral soft tissue and tongue reconstruction, as they allow large amounts of healthy tissue to be transported from sites remote from prior surgical or radiotherapy fields. Commonly used flaps include the radial forearm and anterolateral thigh flaps [14]. However, local and pedicle flaps play important roles in oral and maxillofacial reconstruction, even in the era of free flaps, such as supraclavicular artery island flaps [15, 16], pectoralis major myocutaneous flaps [13, 17], and FSAIF [9, 18–20].

In the present study, hemitongue defects after cancer ablation were reconstructed using FSAIFs, with a success rate of 96.7%. The success rates for oral and maxillofacial defect reconstruction using FSAIF and reverse FSAIF were reported as 95 and 94.4%, respectively [8]. Four patient with FSAIF failure was successfully treated with extensive segmental pectoralis major myocutaneous flap [13].

In an anatomical study, the submental artery was deep relative to the anterior belly of the digastric muscle in 70% of dissections, and superficial to the digastric muscle in the remaining 30% [21]. Several perforator arteries supply the platysma muscle and overlying skin. There are usually two major perforators: one arising proximal to the digastric muscle and the other arising distal to it. Minor perforators pass directly through the anterior belly of the digastric muscle. Flap preparation should include the mandibular hyoid muscle and anterior belly of the digastric muscle to protect the vascular system from accidental injury and ensure its blood supply [8]. Surgical treatment was classified according to the length of surgery: major surgery was defined as that lasting for ≥120 min [22]. In total, 69.7% (85/122) of operations were completed within 120 min, and only 30.3% (37/122) were completed after 120 minutes. The use of FSAIF to repair the tongue defects significantly reduced the length of surgery. The rate of pathologically negative submandibular lymph nodes was 98.4% (120/122). Therefore, FSAIF is a reliable and versatile locoregional flap for the reconstruction of post-resection defects in oral cancer. However, it does not affect locoregional recurrence in clinically node-negative oral cancer patients [20]. In the present study, 68.0% of patients were alive with no evidence of disease, 19.7% were alive with disease, and 12.3% had died due to local recurrence or distant metastases. There was no significant difference in survival among the groups. In addition, 52.5% of patients were able to tolerate a normal diet and 32.8% were able to tolerate a soft diet. Furthermore, 53.3 and 33.6% of patients had normal and intelligible speech, respectively. The aesthetic results were rated as excellent and good in 32.8 and 58.2% of patients, respectively. There was no difference in swallowing, speech, or aesthetic outcome among the three groups. Elderly patients with early disease are suitable for FSAIF because their prognosis is not significantly different from that of younger patients.

Treatment of the neck lymph nodes in early stage OTSCC patients is controversial. Our study enrolled 105 patients treated for neck lymph nodes; the rates of positive neck lymph nodes were 6.3% (4/63) and 41.5% (17/41) in disease stage I and II patients, respectively. The overall risk of isolated regional failure was 6.7% (6/89) and 48.4% (25/60) in stage I and II disease patients, respectively [23]. The results suggested that relatively conservative surgical approaches combined with postoperative radiotherapy should be used for neck node metastasis in elderly patients with OTSCC [20]. Brachytherapy for elderly patients with stage I or II OTSCC was safe, and the success of control of the primary lesion was similar to that in young patients. However, few modalities are suitable to treat neck node metastases. In total, 34.4% (43/125) of the patients were diagnosed with post-brachytherapy neck node metastases, and radical neck dissection was performed in 19.2% (24/125) of the patients [23]. Therefore, relatively conservative surgical approaches combined with postoperative radiotherapy should be used for treating neck node metastases in elderly patients with OTSCC [24]. The salvage surgery for the patients with locoregional recurrence who were reconstructed with FSAIF should be performed.

The comorbidity burden was significantly higher in patients with head and neck squamous cell cancer compared to the general population, and a higher number of comorbidities was associated with increased cancer-related mortality [25]. The Clavien-Dindo system is useful for grading head and neck surgery complications [26]. Our results showed that elderly patients with OTSCC should be managed similar to young patients. Although the ACE-27 index and Clavien-Dindo scores were higher in the elderly than young group, none of the patients developed grade V surgical complications. The Clavien-Dindo grading system can guide the treatment of patients. Patients with grade IIIa complications, such as bleeding and orocutaneous fistulas, were successfully treated with firm pressure or debridement. Patients with grade IIIb complications, such as hemorrhage and flap failure, required urgent exploratory operation to stop the bleeding, and a pedicle flap was used to repair the defects. Patients with grades IVa and IVb complications, including respiratory and cardiovascular diseases, cerebral hemorrhage, and ischemic stroke, were treated in the intensive care unit; they all recovered.

All patients were treated surgically, including via hemiglossectomy, neck dissection, and hemitongue reconstruction using FSAIF. In addition, patients with stage I and II disease underwent ipsilateral selective neck dissection; those with stage III disease underwent radical neck dissection. Patients with T3N1 disease were also treated with adjuvant radiotherapy. The aforementioned modalities are safe and have proven clinical efficacy for young, middle-aged, and elderly patients with early and middle-stage OTSCC. A multicenter retrospective analysis revealed the clinicopathological features and prognosis of OTSCC were similar in adolescent and young adult patients and elderly patients; there was no significant difference in overall survival between patients who underwent elective neck dissection and those who underwent therapeutic neck dissection in adolescent and young adult and control groups; the indication for elective neck dissection in adolescent and young adult patients with clinical N0 OTSCC is similar to that for elderly patients [27].

FSAIF requires a shorter operation time and hospital stay, and has comparable perioperative outcomes, to free tissue transfer. In addition, flap use was associated with significantly shorter operation times and hospital stays, satisfactory aesthetic outcomes, no increase in the local tumor recurrence rate, fewer perioperative complications, and prolonged survival [28–31]. However, the use of FSAIF did not affect locoregional recurrence in clinically node-negative OTSCC patients [20], although the harvested flap area was significantly smaller.

FSAIF still has some disadvantages. There are anatomical variations in submental vessels, which require higher operating requirements for physicians; The submandibular lymph nodes should be carefully removed and subjected to the rapid pathological diagnosis during flap elevation; Once it is found that there is lymph node metastasis but no extranodal extension, the flap can be used on the premise of thorough radical neck dissection, and adjuvant treatment such as radiotherapy and chemotherapy can be performed following surgery; The contraindication is cervical lymph node metastasis of OTSCC and extranodal extension, which should be repaired with other schemes.

The FSAIF, which is a locoregional flap, is simple, safe, and reliable for the reconstruction of hemitongue defects after cancer ablation in young, middle-aged, and elderly patients with early and middle-stage OTSCC.

## Data Availability

Data sharing is not applicable to this article as no datasets were generated or analysed during the current study.
